# Factors Affecting Hemodialysis Patients' Satisfaction with Their Dialysis Therapy

**DOI:** 10.4061/2010/342901

**Published:** 2010-10-25

**Authors:** M. Al Eissa, M. Al Sulaiman, M. Jondeby, A. Karkar, M. Barahmein, F. A. M. Shaheen, A. Al Sayyari

**Affiliations:** ^1^College of Medicine, King Saud Bin Abdulaziz University for Health Sciences, Riyadh 11426, Saudi Arabia; ^2^Department of Nephrology, Riyadh Armed Forces Hospital, Riyadh 11159, Saudi Arabia; ^3^Riyadh Armed Forces Hospital, Riyadh 11159, Saudi Arabia; ^4^Kanoo Dialysis Unit, Dammam, Saudi Arabia; ^5^Dialysis Unit King Fahd Specialist Hospital, Buraidah, Saudi Arabia; ^6^Saudi Center for Organ Transplantation, Riyadh 11417, Saudi Arabia; ^7^King Saud Bin Abdulaziz University for Health Sciences, P.O. Box 22490, Riyadh 11426, Saudi Arabia

## Abstract

*Aim*. To assess the degree of satisfaction among hemodialysis patients and the factors influencing this satisfaction. *Methods*. Patients were recruited from 3 Saudi dialysis centers. Demographic data was collected. Using 1 to 10 Likert scale, the patients were asked to rate the *overall satisfaction* with, and *the overall impact* of, their dialysis therapy on their lives and to rate the effect of the dialysis therapy on 15 qualities of life domains. *Results*. 322 patients were recruited (72.6% of the total eligible patients). The mean age was 51.7 years (±15.4); 58% have been on dialysis for >3 years. The mean Charlson Comorbidity Index was 3.2 (±2), and Kt/V was 1.3 (±0.44). The mean satisfaction score was (7.41 ± 2.75) and the mean score of the impact of the dialysis on the patients' lives was 5.32 ± 2.55. Male patients reported worse effect of dialysis on family life, social life, energy, and appetite. Longer period since the commencement of dialysis was associated with adverse effect on finances and energy. Lower level of education was associated with worse dialysis effect on stress, overall health, sexual life, hobbies, and exercise ability. *Conclusion*. The level of satisfaction is affected by gender, duration on dialysis, educational level, and standard of care given.

## 1. Introduction

Patients' quality of life (QOL) and satisfaction assessment are becoming increasingly important in health care delivery [1, 2]. There is evidence that better QOL and patient satisfaction might be associated with better medical outcome including reduced hospitalization [3] and reduced mortality [4]. Recently more attention has been paid to patient preferences in various renal replacement therapy modalities [5–7].

We studied satisfaction among Saudi hemodialysis patients in 3 different dialysis units in 3 different Saudi cities. We used a previously validated questionnaire [8] which was translated into Arabic; we studied the impact of age, dialysis adequacy, and Charlson Comorbidity Index (CCI) [9] on satisfaction scores in different QOL domains.

## 2. Method

Patients were recruited from 3 hemodialysis units in 3 different Saudi cities (Riyadh, Dammam, and Buraidah). Demographic data on age, cause of renal failure, educational level, gender duration (in months) on dialysis as well as Charlson Comorbidity Index and Kt/V were collected. 

All questionnaires were distributed by one investigator (M. E.), who was not affiliated with any of the dialysis units. Questionnaires were completed by the patients during their dialysis sessions. M. E. was available to answer any queries by the patients.

The questionnaire used was developed by Juergensen et al. [8]. This was translated into Arabic and checked by 3 Arab nephrologists and tested in 30 patients before being used. 

To be eligible for inclusion in the study, the patient must be over 16 years of age and has been on dialysis for at least 6 months, be medically stable without acute medical problems for a minimum of 3 months before the study, and be able to understand and answer the questionnaires. 

Data was recorded on age, gender, duration on dialysis, education level, Charlson Comorbidity Index, cause of renal failure, and Kt/V. 

Using 1 to 10 Likert scale the patients were asked to rate their *overall satisfaction *with, and *the overall impact *of, their dialysis therapy on their lives as well as to rate the effect of dialysis on 15 domains that impact quality of life (including overall health, stress level, family life, social life, independence, finances, mood, religion/spirituality, sex life, energy level, recreation/ hobbies, exercise ability, living arrangements, appetite, and body image). The patients were also asked to list 3 positive and 3 negative effects of dialysis.

 Only patients over 16 years of age who are medically stable without acute medical problems for a minimum of 2 months before the study were included. They, also, had to be able to understand and answer the questionnaires. 

Means were compared using two-sided *t-test, and the impact of CCI *on satisfaction scores was assessed using Pearson correlation coefficient. The effects of different categorical factors (above and below median) of age, gender, comorbidity index (CCI), education, and duration since commencement of dialysis as well as of the presence or absence of diabetes, marital status, and employment status on scores for different satisfaction domains were assessed using independent two-tailed *t*-test. 

Ethical approval was obtained from the Research and Ethical Committee of the College of Medicine King Saud Bin Abdulaziz University for Health Sciences.

## 3. Results

322 patients (72.6% of the total eligible patients) were included from 3 dialysis centers. These were selected randomly and did not differ in any significant parameter from those not enrolled. The mean age was 51.0 years (±15.5), and 62.2% were male. 57.7% of the patients were on dialysis for more than 3 years, 57.1% had less than 4 years of education, and only 4% entered university. Diabetic nephropathy was the cause of renal failure in 25.4%. The mean Charlson Comorbidity Index was 3.4 (±1.6), and the mean Kt/V was 1.3 (±0.4) (Table 1).

The mean overall “dialysis satisfaction” score for all the 3 dialysis centers together was 7.41 ± 2.75. When calculated for each city separately, Riyadh, Dammam, and Buraidah, the “dialysis satisfaction” scores were 9.0 (2.0), 6.5 (2.4), and 4.7 (2.2), respectively (*P* = .0001) (Table 2).

On the other hand, when asked about the “effect of the dialysis therapy on their lives,” the mean overall score was lower (5.32 ± 2.55). For Riyadh, Dammam, and Buraidah, the scores were 6.2 (2.6), 4.5 (2.3), and 4.6 (92) (i.e., mean and standard deviation), respectively, (*P* < .002) (Table 2).

Table 3 compares the mean age, Kt/V, and Comorbidity Index (CCI) in the 3 cities in Saudi Arabia.

Gender, years of education, and duration on dialysis (duration since dialysis was commenced (in months), did not have any effect on the level of dialysis satisfaction. The years of education had an impact on or dialysis effect on life with education of less than 3 years being associated with better effect (*P* = .03). However neither gender nor duration on dialysis had any impact on or dialysis effect on life) (Table 4).

The mean overall scores in all the other 15 QOL domains were similar in Riyadh and USA (5.62 (1.99) and 5.96 (1) resp., *P* = .3) (Table 5). However these scores were much lower in Dammam and Buraidah patients (3.78 (1.36) and 3.83 (1.73), resp., *P* < .002) Nevertheless, Riyadh's patients scored higher than USA patients in these areas: global satisfaction, family life, social life, spirituality, and finances.

The worse scores among the 15 QOL domains tested were seen with the effect of dialysis on stress (3.18 ± 2.31), on sexual life (2.71 ± 2.41), on exercise ability (2.25 ± 2.08), and on hobbies (2.81 ± 2.39). By contrast, the least adverse effect of dialysis was seen on the practice of daily prayers, (7.84 ± 2.64  ), on family (5.73 ± 3.04), and social life (5.7 ± 2.9) (Table 6).

When analyzing the impact of duration of dialysis on the 15 QOL domains, we found that duration of dialysis of more than 3 years was associated with more stress and worse financial burden. 

More years of education had a significant positive effect on 7 of the 15 QOL domains (stress, mode, overall health, sexual life, energy, hobbies, and exercise). 

Patients from the Riyadh center scored higher than the patients from Dammam and Buraidah in all but two QOL domains. The exceptions being body image and stress level in both of which Buraidah patients had higher scores (Table 7).

## 4. Discussion

The mean overall dialysis satisfaction score for all the 3 dialysis centers was 7.41 ± 2.75. It was noted to be highest in Riyadh patients (9.0 (2.0)) followed by Dammam patients (6.5 (2.4)) and Buraidah (4.7 (2.2)) (*P* < .0001). 

The mean overall score for the “effect of the dialysis therapy on their life generally” was 5.32 ± 2.55. Again, Riyadh patients had significantly higher score (6.2 (2.6)), than Dammam and Buraidah patients 4.6 (2.3) (i.e., mean and standard deviation) and 4.6 (0 92), resp.,) (*P* < .002).

It is not clear why we have such big differences between the scores from Riyadh on the hand and those from Dammam and Buraidah on the other. This might be related to the more favorable staffing to patient ratio as well as more advanced supporting services—including social services—in Riyadh.

It is worth noting that the results obtained in Riyadh patients in terms of “overall dialysis satisfaction” and “effect of the dialysis therapy on their life generally” were similar to those reported in USA patients [8]. It should be noted that, as with our study, the patients enrolled in the USA study were all adults who have been on dialysis for at least 6 months and were free from acute illness for at least 2 moths. Additionally, as in our patients the questionnaires were completed by the patients during the dialysis session, and all questionnaires were distributed by one investigator, who was not affiliated with the dialysis unit. It was also noteworthy that the mean overall scores in all the other 15 QOL domains were similar in Riyadh and USA (5.62 (1.99) and 5.96 (1), resp., *P* = .3). However these scores were much lower in Dammam and Buraidah patients (3.78 (1.36) and 3.83 (1.73), resp., *P* < .002). Nevertheless, Riyadh's patients scored higher than USA patients in the areas of global satisfaction, family life, social life, spirituality, and finances. These findings are consistent with previous reports that within the Saudi society, family's financial and social support specially for ill family members is strong [10]. It is also in keeping with the high spirituality level of Saudis [11]. 

Among the Saudi patients, the worse scores were seen with the effect of dialysis on stress (3.18 ± 2.31), on sexual life (2.71 ± 2.41), on exercise ability (2.25 ± 2.08), and on hobbies (2.81 ± 2.39). This finding is in keeping with known adverse effects of renal failure and dialysis on these quality of life indicators [3]. By contrast, the least adverse effect of dialysis was seen on the practice of daily prayers (7.84 ± 2.64), on family (5.73 ± 3.04) and social life (5.7 ± 2.9). This gain is in keeping with the known emphasis of social, spiritual, and family life in the Saudi society [12, 13].

When analyzing the impact of duration since the commencement of dialysis on the 15 QOL domains, we found that duration of more than 3 years was associated with more stress and worse financial burden which is perhaps not surprising given the impact of long-standing dialysis on employability [14].

More years of education had a significant positive effect on 7 of the 15 QOL domains (stress, mode, overall health, sexual life, energy, hobbies, and exercise).

Patients from the Riyadh center scored higher than the patients from Dammam and Buraidah in all 15 but two QOL domains. The exceptions being body image and stress level in both of which Buraidah patients had higher score.

These discrepancies cannot be explained by differences in age or Charlson Comorbidity indices. However they might be due to the higher Kt/V in Riyadh patients (*P* < .0001). Alternatively they might partly be explained by Riyadh patients being more survey question compliant and “eager to please.” We have found evidence of this in a previous study we carried out [15].

In a previous study we did in Saudi dialysis patients using KDQOL-SF36, we also found that the domains viewed positively (score >80) were “patient satisfaction,” “dialysis staff encouragement,” and “quality of social interaction.” These scores were not affected by level of education, age, duration on dialysis, or cause of renal failure [14]. 

The main negative effects of dialysis reported by our patients were fatigue, dizziness, and boredom, and the main positive effects of dialysis reported were improved energy and breathing.

## 5. Conclusions

The level of satisfaction was different in the three cities studied. This might be related to the different degrees of dialysis adequacy or to survey response characteristics. 

The least adverse effect of dialysis was seen on the practice of daily prayers and social life. This is in keeping with the known emphasis of social, spiritual, and family life in the Saudi society. 

Male patients reported worse effect of dialysis on family life, social life, energy, appetite than females. Longer dialysis duration was associated with adverse effect on finances, energy, and living arrangement. Lower level of education was associated with worse dialysis effect on stress, overall health, sexual life, hobbies, and exercise ability. 

##  Conflict of Interests

The authors declare that they have no competing interests.

##  Disclosure

Al Eissa wrote up the proposal selected the respondents, collected and entered the data at King Abdulaziz Medical City, Riyadh. Al Sulaiman contributed to the design of the research project, Jondeby selected the respondents, collected and entered the data at Riyadh Armed Forces Hospital, Riyadh. Karkar selected the respondents, collected and entered the data at Kano Center, Dammam. Barahmein selected the respondents, collected and entered the data at King Fahd Specialist Hospital, Buraidah. Shaheen contributed to writing the proposal, did the analysis and writing of the paper. Al Sayyari contributed to writing the proposal, did the analysis and writing of the paper.

## Figures and Tables

**Table 1:** Demographic data.

Age	51.0 (±15.5)
Males (%)	62.2%
Level of education	
<4 years of education	57.1%
University level	4%
Duration on dialysis >3 years	57.7%
CKD due to diabetic nephropathy	25.4%
Mean Kt/V	1.3 (±0.4)
Mean CCI	3.4 (±1.6)

**Table 2:** Global satisfaction and global impact of therapy scores of patients from Riyadh, Dammam, and Buraidah.

	Riyadh (R)	Dammam (D)	Buraidah (B)	P (R versus D)	P ( R versus B)	P (D versus B)
Global satisfaction	9	6.5	4.7	.0001	.0001	.0001
Global impact of therapy	6.2	4.6	4.6	.0001	.002	.0001

**Table 3:** Age, Kt/V, and Comorbidity Index in the 3 cities in Saudi Arabia.

	Riyadh	Dammam	Buraidah	*P* value
Age	51.8	51	48	0.7
Kt/V	1.5	.99	1.17	.0001
CCI	3.48	3.4	3.2	.9

**Table 4:** Comparing scores in the two global scores by gender, duration on dialysis, and years of education.

	Level of dialysis satisfaction	*P*	Dialysis effect on life	*P*
Male	7.31 (2.8)		5.19 (2.5)	
Female	7.53 (2.7)	.49	5.52 (2.7)	.28
>3 years on dialysis	7.38 (2.8)		5.06 (2.6)	
<3 years on dialysis	7.42 (2.6)	.9	5.68 (2.5)	.03
Education >4 years	7.61 (2.8)		5.26 (2.5)	
Education <4 years	7.27 (2.7)	.29	5.33 (2.6)	.8

**Table 5:** Comparing overall mean scores in Riyadh, Dammam, Buraidah, and USA.

	USA	Riyadh	Buraidah	Dammam
Mean	5.96	5.62	3.83	3.78
Std. Deviation	1.00	1.99	1.73	1.36
*P* value (compared to USA)	1	.3	.0001	.0001
*P* value (compared to Riyadh)	.3	1	.002	.0001
*P* value (compared to Buraidah)	.0001	.002	1	.9

**Table 6:** Mean combined scores for all the QOL domains (in descending order).

	Mean	STD
Effect on praying	7.84	2.64
Effect on family life	5.73	3.04
Effect on social life	5.70	2.91
Effect on appetite	5.38	2.44
Effect on finance	5.37	3.59
Effect on independency	5.25	2.42
Effect on living arrangements	5.08	2.25
Effect on overall health	4.67	2.80
Effect on body image	4.27	2.45
Effect on effect on mood	3.67	2.52
Effect on effect on energy	3.43	2.46
Effect on stress level	3.18	2.13
Effect on effect on hobbies	2.81	2.39
Effect on sexual life	2.71	2.41
Effect on exercise ability	2.25	2.08

**Table 7:** Comparing scores in the 15 QOL domains by Center.

	Riyadh		Dammam		Buraidah	
	Mean	STD	Mean	STD	Mean	STD
Dialysis effect on life	6.16	2.64	4.56	2.29	4.62	2.01
Stress level	3.51	1.91	2.12	1.78	3.78	2.38
Overall health	6.15	2.52	2.65	2.11	4.02	2.26
Family life	7.55	2.73	3.77	2.25	4.37	2.14
Social life	7.40	2.66	4.24	2.35	4.14	2.19
Independency	6.12	2.10	4.56	2.31	3.94	2.40
Finance	6.12	2.77	5.10	5.03	4.00	2.33
Effect on mood	4.89	2.16	2.78	2.25	1.63	1.82
Effect on praying	9.70	1.09	5.70	2.49	8.45	1.22
Effect on sexual life	3.13	2.53	2.35	2.11	1.43	1.31
Effect on energy	4.60	2.44	2.59	2.04	1.52	1.45
Effect on hobbies	3.02	2.68	2.41	1.93	2.12	2.05
Exercise ability	2.42	2.32	1.99	1.61	1.81	1.79
Living arrangements	5.68	1.91	4.48	2.84	4.77	1.91
Appetite	5.57	2.48	5.29	2.82	5.31	1.35
Body image	4.49	2.42	3.82	2.71	4.71	1.92
Mean	5.41		3.65		3.79	
